# Carotid Body Tumor: Incidental Discovery and Diagnosis

**DOI:** 10.7759/cureus.75074

**Published:** 2024-12-04

**Authors:** Sidhartha G Senapati, Lakshmi Kattamuri, Abhizith Deoker

**Affiliations:** 1 Internal Medicine, Texas Tech University Health Sciences Center, El Paso, USA

**Keywords:** atrial fibrillation, carotid body tumors, neuroendocrine neoplasms, paraganglioma, pheochromocytoma

## Abstract

Carotid body tumors (CBTs), rare neuroendocrine neoplasms near the carotid bifurcation, are mostly asymptomatic but may cause discomfort and autonomic dysfunction. Computed tomography angiography (CTA) is used for diagnosis, eliminating the need for a biopsy to avoid the risk of hemorrhage. Surgical excision is the preferred treatment, while radiotherapy is an option when surgery is impractical. A 75-year-old woman with diabetes and hypertension presented to the emergency room (ER) with nausea, vomiting, and severe headache. Her blood pressure was 196/134 mmHg, her heart rate was 140 beats per minute (bpm), and her electrocardiogram (EKG) showed atrial fibrillation (AF). Physical examination revealed elevated jugular venous pressure. Preliminary lab investigations were normal. CT of the head, followed by an MRI of the neck with and without contrast, showed a stable 38 x 53 x 15 mm mass in the left side of the neck consistent with carotid body paraganglioma. A hormonal workup indicated elevated metanephrines, ruling out adrenal tumors. An abdominal CT scan showed no adrenal tumors. Due to her age and comorbidities, radiotherapy was planned for the stable mass. This article discusses the diagnosis of a rare, slow-growing carotid body tumor that requires thorough assessment, including patient history and radiologic evaluation.

## Introduction

Carotid body tumors (CBTs), also known as paragangliomas or chemodectomas, are uncommon neuroendocrine neoplasms that develop in glomus cells generated from the embryonic neural crest close to the carotid bifurcation. CBTs are reported to occur in 1-2 individuals per 100,000 people [1]. Most of these tumors are asymptomatic and are typically discovered incidentally during radiological imaging or clinical examination. However, in symptomatic cases, discomfort, dysphagia, and autonomic dysfunction are the most commonly reported symptoms. The diagnosis of paragangliomas is primarily based on imaging, with computed tomography angiography (CTA) being the examination of choice. If a paraganglioma is suspected, a biopsy is contraindicated due to the high risk of hemorrhage [2,3]. The preferred treatment is surgical excision, although embolization may also be performed to reduce tumor size and minimize bleeding during surgery [4]. In cases where surgery is not feasible or necessary, radiotherapy may be considered [4]. Through this article, we aim to contribute to the collective understanding and knowledge surrounding the incidental discovery and diagnosis of this rare, slow-growing CBT.

This article was previously posted as a preprint on the Research Square preprint server on 17 May 2024 [5].

## Case presentation

A 75-year-old woman with a 34-year history of diabetes and hypertension presented to the emergency room (ER) after experiencing multiple episodes of nausea and vomiting over the past 24 hours. She also reported a severe headache but denied any chest pain, abdominal pain, diarrhea, or dysuria. Upon admission, her blood pressure was 196/134 mmHg, and her heart rate was 140 beats per minute (bpm). The electrocardiogram (EKG) revealed atrial fibrillation (AF) with a rapid ventricular rate. She denied any prior history of AF and reported being compliant with all her medications with regular follow-ups with her primary care physician. She also reported no history of fever, cough, weight loss, anorexia, night sweats, diaphoresis, palpitations, dizziness, lightheadedness, or syncopal episodes. Physical examination showed elevated jugular venous pressure (JVP) with normal cardiac auscultation, and the rest of the systemic examinations were unremarkable.

Investigations

**Figure 1:**
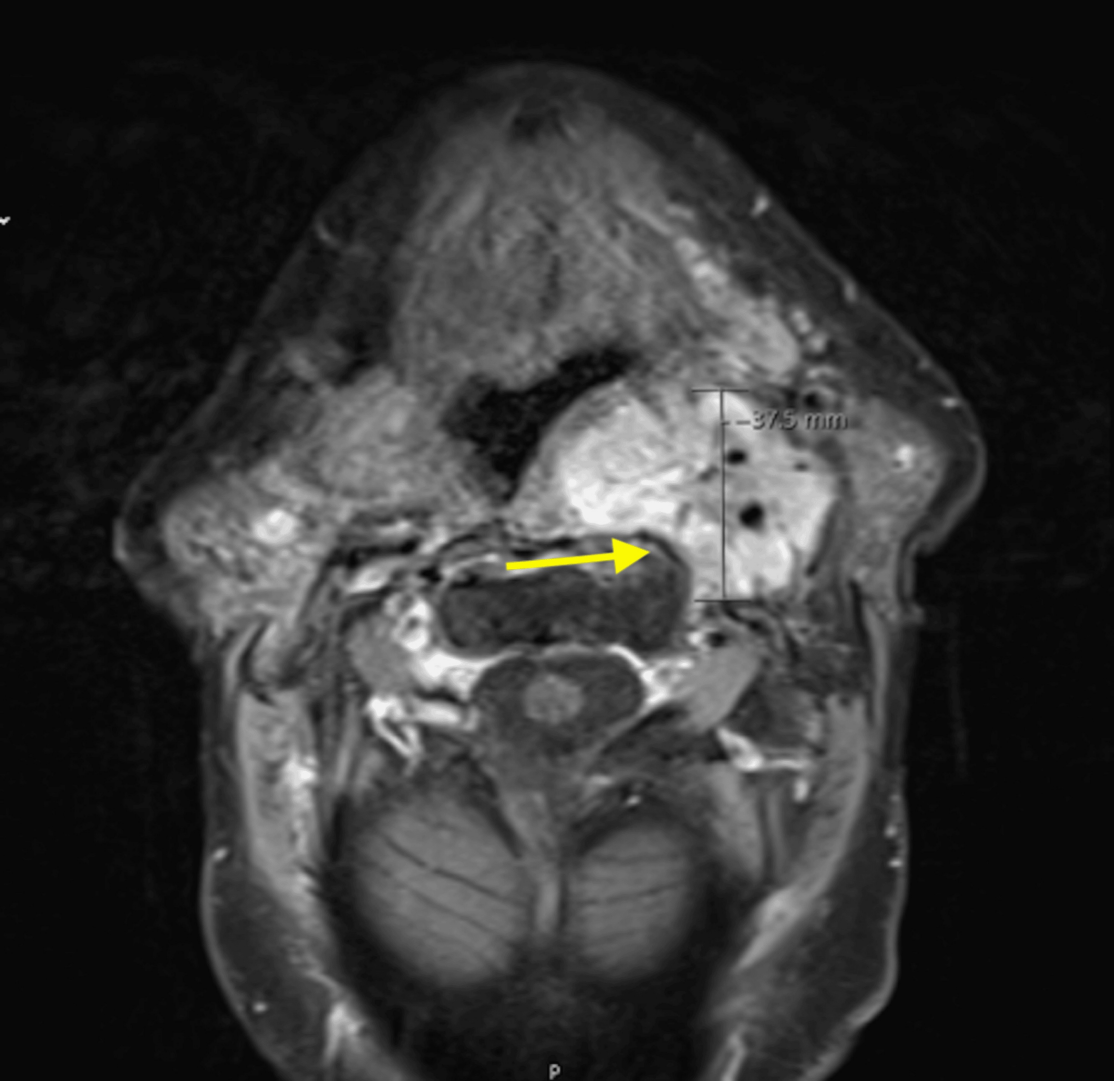
T1-weighted gadolinium-enhanced MRI sequence of the neck showing a mass on the left side (as indicated by the yellow arrow).

Initial blood tests, including a complete blood count, renal function tests, liver function tests, and thyroid function tests (Table 1) were all within the normal limits. Additionally, a urine toxicology screen was negative for cannabinoids, amphetamines, or cocaine. The chest X-ray revealed no acute abnormalities. Similarly, the contrast-enhanced CT scan of the thorax and abdomen was normal. The CT scan of the head with contrast also indicated no acute abnormalities, except for an unclear submandibular mass. Further evaluation with a contrast-enhanced CT scan of the neck and an MRI of the neck with and without contrast revealed a stable mass measuring 38 x 53 x 15 mm on the left side, consistent with a carotid body paraganglioma (CBP) (Figure 1). This mass had remained stable in size compared to the previous examination seven years ago. Given the patient's newly diagnosed AF and hypertension, concern arose about a functionally active tumor. The hormonal evaluation showed elevated levels of spot plasma and urine metanephrines, while an abdominal CT scan showed no adrenal tumors.

**Table 1:** Laboratory workup.

Peripheral blood			Reference range
White blood cell (x 10^3^/µL)	8.95		4.50-11.00
Red blood cell (x 10^6^/µL)	3.44		3.5-4.5
Hemoglobin (g/dL)	11.2		12.00-15.00
Hematocrit (%)	35.3		36.0-47.0
Platelets (x 10^3^/ µL)	430		150-450
Coagulation study			
Prothrombin time (seconds)	15.1		11.8-14.8
Activated partial thromboplastin time (APTT) (seconds)	22.0		23.3-38.6
Blood chemistry			
Total protein (g/dL)	6.6		6.3-8.2
Albumin (g/dL)	4.0		3.5-5.0
Total bilirubin (mg/dL)	0.4		0.2-1.3
Aspartate aminotransferase (AST) (U/L)	16		14-36
Alanine aminotransferase (ALT) (U/L)	29		0-35
Alkaline phosphatase (ALP) (U/L)	47		38-126
Blood urea nitrogen (mg/dL)	12		7-17
Creatinine (mg/dL)	0.6		0.52-1.04
Sodium (Na) (mmol/L)	137		135-145
Potassium (K) (mmol/L)	4.0		3.5-5.1
Chlorine (Cl) (mmol/L)	103		98-107
Calcium (Ca) (mg/dL)	8.8		8.4-10.2
Thyroid-stimulating hormone (TSH) (µIU/mL)	2.12		0.465-4.68
Urine drug screen			
Cannabinoids	Negative		-
Barbiturates	Negative		-
Amphetamine	Negative		-
Benzo	Negative		-
Cocaine	Negative		-
Opiates	Negative		-
Complements	Positive		-
Urine metanephrines			
Metanephrines random (mcg/g creatinine)	456		21-153
Normetanephrine random (mcg/g creatinine)	1349		108-524
Plasma metanephrines			
Metanephrines, free (pg/mL)		96	< 0R = 57
Normetanephrine, free (pg/mL)		218	

Treatment

Her home medications of lisinopril 40 mg and amlodipine 10 mg were resumed. Despite being on two antihypertensives, her blood pressure remained at 180/94 mmHg, with a heart rate of 140 bpm. She was initially treated with intravenous (IV) metoprolol, and once her blood pressure and heart rate stabilized, she was switched to oral metoprolol. A conservative dosage of 12.5 mg metoprolol was given twice daily, with gradual increases based on her tolerance. Nausea was managed appropriately, and her electrolytes were optimized, allowing for diet advancements as tolerated. Given her age and associated comorbidities, radiotherapy was planned; however, the patient declined the treatment.

Outcome

Once her vital signs stabilized and her nausea and vomiting subsided, there was significant improvement in her overall clinical condition. Metoprolol effectively controlled her heart rate and blood pressure, allowing for her discharge.

## Discussion

A paraganglioma is a type of tumor that arises from paraganglia, which are clusters of neuroendocrine cells derived from neural crest cells. These tumors commonly arise in areas with abundant paraganglia, such as the carotid body, the vagus nerve in the head and neck, the jugular foramen, the middle ear, the aortopulmonary window, and the organ of Zuckerkandl [6]. Among these, the carotid body is the most significant as it contains the largest concentration of paraganglia in the head and neck. This structure functions as a chemoreceptor, primarily monitoring oxygen levels in the blood. The carotid body helps regulate vital autonomic functions by initiating a sympathetic nervous system response to detect arterial hypoxemia. Typically, the carotid body measures around 3-5 mm, though it can grow over 8 cm in response to persistent hypoxic conditions [4,7]. As a chemoreceptor, the carotid body responds to acidosis, hypoxia, and hypercapnia, playing a vital role in regulating blood pressure, heart rate, respiration, and blood temperature through increased sympathetic nervous system activity [8]. 

CBTs account for approximately 65% of all paragangliomas in the head and neck [9]. These tumors can manifest at any age, though they are most commonly seen in individuals aged 50-70 years, with a range from 18 to 94 years. Women are slightly more affected than men, with a male-to-female ratio of 1:1.9 [10]. CBTs are typically sporadic, with around 25% having a hereditary basis due to mutations in succinate dehydrogenase genes, including SDHD, SDHB, and SDHC [4]. Genetic mutations are the only identified risk factor for the development of CBTs, aside from hypoxic stimulation, and familial cases often present with bilateral CBTs at a higher frequency [10]. 

CBTs and paragangliomas in the head and neck are typically painless, slow-growing tumors that often remain undetected for years before patients seek medical attention. The tumors can grow to significant sizes and may exhibit infiltrative growth, with local recurrence posing a risk of fatality. Five percent of paragangliomas secrete catecholamines (dopamine, epinephrine, and norepinephrine), with hypertension being the most common manifestation. This hypertension can be continuous, intermittent, or paroxysmal [11]. The classic triad of headache, sweating, and palpitations occurs in 40% of cases [12]. Dopamine-producing paragangliomas, which are rare, can present with normal blood pressure or even hypotension. Limited case reports suggest that CBTs, like pheochromocytoma, may present with AF. In cases of elevated catecholamine levels, additional imaging is recommended to exclude intra-abdominal tumors. Malignancy rates range from 10% to 50%. Histologic features indicating malignancy include necrosis, extensive capsular or vascular invasion, increased mitotic activity, atypical mitotic figures, loss of a well-differentiated zellballen pattern, reduction in the S-100 positive sustentacular cell population, and tumor cell spindling [13]. 

CBTs are most commonly discovered incidentally during imaging studies or through clinical assessments. The most effective screening method for CBTs is a color-flow carotid duplex, where CBTs are identified by a well-defined hypoechoic mass causing the splaying of the internal carotid artery (ICA) and external carotid artery (ECA). Color Doppler imaging reveals hypervascularity within the lesion with a low-resistance flow pattern. Surgical removal depends on the tumor's relationship with the artery bifurcation and its proximity to cranial nerves, which can be accurately assessed through CTA or MR angiography (MRA). MRI is often more precise than CT due to its lack of ionizing radiation. Dynamic contrast-enhanced MRI and diffusion-weighted imaging (DWI) are particularly effective in distinguishing paragangliomas from other benign neoplastic tumors [14]. 

Radical surgical resection is performed to prevent malignant transformation [3]. The preoperative adrenergic blockade is recommended, while preoperative embolization remains controversial due to potential risks [15]. Although embolization may be considered for large tumors, it increases the risk of transient ischemic attacks and ischemic strokes [16,17]. Radiotherapy, recommended for patients with various medical conditions at risk during general anesthesia, has been shown to reduce tumor size or halt its growth. 

This case involves a 75-year-old woman with carotid body paraganglioma (CBP), complicated by new-onset AF and severe hypertension. While Hoang et al. focused on the presentation and surgical management of CBTs, this case offers novel insights into the association between paragangliomas and cardiovascular complications like AF, which are rarely documented [4]. This case discusses the successful management of AF and severe hypertension using IV and oral metoprolol, illustrating the importance of careful cardiovascular management in patients with functionally active paragangliomas. This approach, particularly in an older patient with multiple comorbidities, adds value to the literature by focusing on the nonsurgical management of cardiovascular symptoms in CBT patients.

Compared to the case review by Hoang et al. [4], which focused on more typical symptoms of CBTs (e.g., neck mass, dysphagia, and hoarseness), this case demonstrates an atypical presentation without overt neck swelling, hoarseness, or swallowing issues. This challenges the assumption that CBTs present predominantly with local symptoms, expanding the understanding of the disease’s clinical spectrum. This report contributes to the literature by documenting the occurrence of AF, which has not been commonly emphasized in previous case studies of paragangliomas, including the study by Hoang et al. [4]. The link between paragangliomas and secondary cardiovascular complications, particularly in the absence of adrenal tumors, is an emerging area that this case explores, offering an update on potential complications that should be monitored.

The detailed hormonal workup and the discovery of elevated plasma and urine metanephrines underscore the importance of assessing functional activity in suspected paragangliomas. This reinforces the need for comprehensive hormonal evaluation, an aspect that may not have been emphasized in earlier reports. This finding is particularly relevant for clinicians when considering differential diagnoses for patients with new-onset AF and uncontrolled hypertension.

## Conclusions

This article shares our insights and experiences in managing CBTs, which are rare, slow-growing, hypervascular neuroendocrine tumors with the potential for malignant transformation. Diagnosis typically involves a thorough assessment, including patient history, physical examinations, and radiological studies. This report presents an older patient with CBP and new-onset AF, highlighting the need for further research into the cardiovascular manifestations of paragangliomas.
